# Magnetic carbon nanotubes for self-regulating temperature hyperthermia †

**DOI:** 10.1039/c7ra13256e

**Published:** 2018-03-27

**Authors:** Xudong Zuo, Chengwei Wu, Wei Zhang, Wei Gao

**Affiliations:** State Key Laboratory of Structural Analysis for Industrial Equipment, Department of Engineering Mechanics, Dalian University of Technology Dalian 116024 P. R. China wei.zhang@dlut.edu.cn; Department of Chemical and Materials Engineering, The University of Auckland Auckland 1142 New Zealand

## Abstract

Magnetic hyperthermia can enhance the anti-tumor effects of chemotherapy. As carbon nanotubes are ideal drug carriers for chemotherapy, their combination with magnetic nanoparticles provides a novel chance for multi-modal thermo-chemotherapy. Most related work focuses on attaching Fe_3_O_4_ nanoparticles on carbon nanotubes, however the hyperthermia temperature for this combination can not be self-regulated due to the high Curie temperature of Fe_3_O_4_. In this work, magnetic Zn_0.54_Co_0.46_Cr_0.6_Fe_1.4_O_4_ nanoparticles with low Curie temperature were attached onto carbon nanotubes to obtain the magnetic carbon nanotubes. The morphology, formation mechanism, magnetic properties, heat generation ability and cytotoxicity of the magnetic carbon nanotubes were investigated. These magnetic carbon nanotubes show a Curie temperature of 43 °C and a self-regulating temperature at 42.7 °C under clinically applied magnetic field conditions (frequency: 100 kHz, intensity: 200 Oe). The evaluation of *in vitro* cytotoxicity suggests no obvious toxicity effects under the concentrations of 6.25 μg ml^−1^ to 100 μg ml^−1^. This study proposed a methodology for the bespoke synthesis of magnetic carbon nanotubes with a low Curie temperature for self-regulating magnetic hyperthermia, which may be used for further research on loading drugs for multi-modal cancer therapy.

## Abstract 2

Magnetic carbon nanotubes with low Curie temperature are synthesized for self-regulating temperature in hyperthermia.

## Graphical abstract

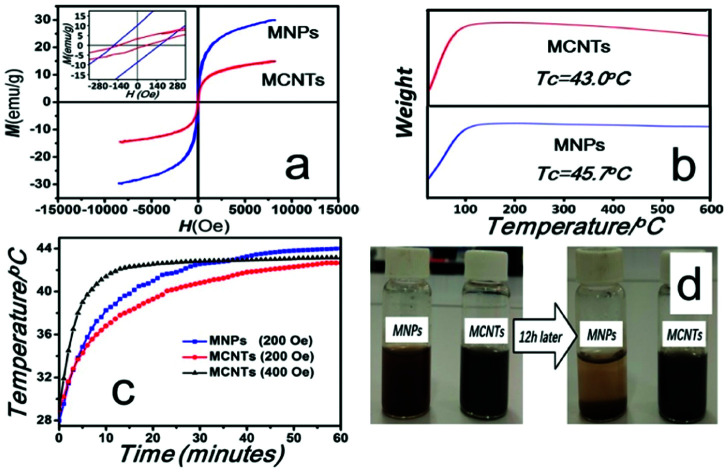

## Introduction

Magnetic nanoparticle-based hyperthermia is considered as a green method for treating cancer due to its low side effects.^1–3^ Basically, magnetic nanoparticles are injected into the tumor region and generate heat upon the application of an external alternating magnetic field. Since normal cells possess higher heat resistance than tumor cells, the tumor cells can be killed selectively without affecting adjacent normal tissue if the temperature can be maintained at 42–45 °C.^4–6^

Carbon nanotubes (CNTs) are well ordered, hollow graphitic materials with high aspect ratio.^7–9^ Due to the ability to move easily among tissues and compartments of body and come across the membrane into the cell *via* endocytosis and diffusion, CNTs are considered as perfect carriers for drugs, nucleic acid and imaging agents for targeting therapy.^10–12^ The large surface area of CNTs, together with their hollow structure, enables them to be loaded with a large quantity of drug molecules, which can effectively prolong the circulation time of drug molecules in blood and enhance cellular uptake of the drug by cancer cells.^13–16^ Graphic surface of CNTs can also be modified in order to graft specific antibodies, avoiding immune action and reducing the possibilities of undesired cytotoxicity.^17^ These fascinating properties make CNTs emerge as promising therapy-enhancing nanomaterials.

Hyperthermia can enhance the anti-tumor effects of chemotherapy by increasing the uptake of carcinostatics into tumor cells and inhibiting the repair of tumor cells.^18,19^ The dose of anti-tumor drugs, therefore, can be decreased by the combination with hyperthermia, and the undesirable side effects of chemotherapy drugs can be also minimized. As CNTs are ideal carriers of drugs, their combination of magnetic nanoparticles may provide a possibility for further research on multi-modal thermos-chemotherapy. However, most of related work focus on attaching Fe_3_O_4_ nanoparticles on CNTs, but the hyperthermia temperature *in vivo* is hard to monitor and control. And a temperature higher than 45 °C may cause damage to normal tissue.^20,21^

Tuning the Curie temperature of magnetic media to a value just above the treatment temperature is considered as an expedient route to control the magnetic hyperthermia temperature and realize self-regulation.^22–25^ Curie temperature is the temperature at which ferromagnetic materials lose their intrinsic permanent magnetic properties and consequently lose their ability to generate heat under alternating magnetic field. The Curie temperature therefore gives an upper limit to the operational temperature for the magnetic media. In the present work, we prepared magnetic nanoparticles (Zn_0.54_Co_0.46_Cr_0.6_Fe_1.4_O_4_) with Curie temperature at 45.7 °C and successfully decorated them on the surface of CNTs through hydrothermal method. Magnetic carbon nanotubes (MCNTs) with Curie temperature at 43 °C were obtained. These MCNTs show a Curie temperature of 43 °C and self-regulating temperature at 42.7 °C under clinically applied magnetic field conditions (frequency: 100 kHz, intensity: 200 Oe), which is suitable for hyperthermia use. Compared with pure nanoparticles, MCNTs have a better dispersive ability to avoid the inhomogeneous temperature distribution caused by the aggregation of nanoparticles. This study proposed a method on the bespoke synthesis of MCNTs with low Curie temperature for self-regulating magnetic hyperthermia, which may be used of further research on loading drugs for multi-modal cancer therapy.

## Experimental

### Chemical synthesis

FeCl_3_·6H_2_O (99%+) and CrCl_3_·6H_2_O (99%+) were obtained from Shantou Xilong Chemical Co. Ltd., China. CoCl_2_·6H_2_O (99%+) and NaOH (96%+) were supplied by Tianjin Bodi Chemical Co. Ltd., China. ZnCl_2_ (98%+) was supplied by Tianjin Damao Chemical Co. Ltd., China. Carboxylic multi-walled CNTs were purchased from Shenzhen Nanotech Port Co. Ltd., China. CrCl_3_·6H_2_O (3.3 mmol), FeCl_3_·6H_2_O (7.7 mmol), CoCl_2_·6H_2_O (2.53 mmol), ZnCl_2_ (2.97 mmol) and carboxylic CNTs (0.75 g) were dissolved in 80 ml deionized water under stirring for 1 h. Then the NaOH solution (0.5 mol L^−1^, 150 ml) was slowly dropped into the metal salts solution with vigorous stirring for 30 min to form the precursor. The precursor was sealed in autoclave and heated to 250 °C (heating rate: 2.3 °C min), maintained for 2 h and then allowed to cool to room temperature. The products were washed with deionized water and ethanol till neutral, and then dried at 60 °C for 6 h in a vacuum drying chamber. Magnetic nanoparticles (MNPs) were also synthesized through the same way without adding carboxylic CNTs for comparison purpose.

### Characterization

The crystal structure was characterized by PANalytical Empyrean X-ray diffractometer (XRD) (Netherlands) with Cu Kα radiation (*λ* = 0.15406 nm). The Raman analysis was conducted on a Renishaw Invia Laser Raman Spectrometer (UK) with a green laser (*λ* = 532 nm) incitation. Fourier transform infrared spectroscopy (FTIR) data were collected on a NEXUS 670 spectrometer. The transmission electron microscope (TEM) images were obtained on a FEI Tecnai G2 F30 (USA). Magnetization curves were recorded at 300 K on a Jilin University JDM-13 vibrating sample magnetometer (China).

The Curie temperature was measured by thermogravimetric analysis (TGA) using Mettler-Toledo TGA 851 (Switzerland) with a Nd–Fe–B magnet (100 × 50 × 5 mm^3^) placed up over the samples about 10 cm. When the temperature is below Curie temperature, the weights of MCNTs recorded by TGA are less than real weights due to the attractive magnetic force between MCNTs and the magnet. When temperature increases to the Curie point, the MCNTs loses their magnetism and the weight becomes the real weight. The transition point is the Curie temperature.

**Fig. 1:**
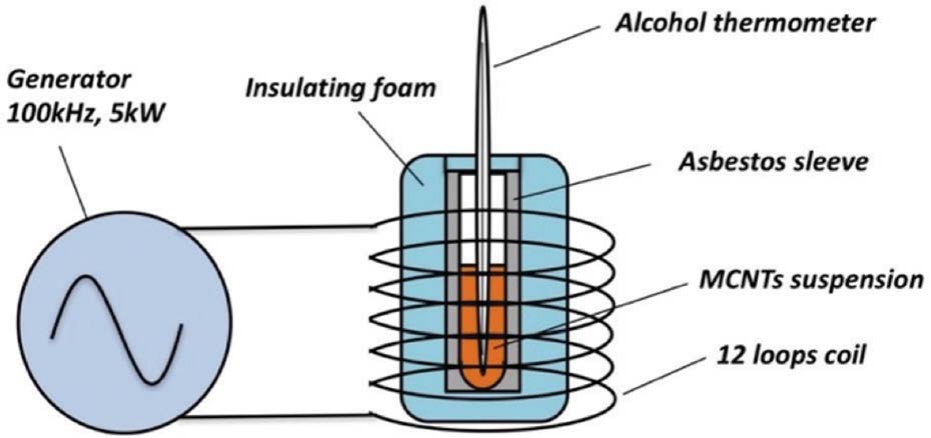
The experimental setup of calorimetric measurements.

The time-dependent temperature curves were determined by calorimetric measurements. The experimental setup is illustrated in Fig. 1. To make the performance of MCNTs under the alternating magnetic field closer to the clinic treatment, the experimental condition was set up as the same as Jordan's clinical treatment.^26,27^ All the MCNTs suspension were dispersed into deionized water with the same concentration (112 mg ml^−1^), and the frequency and intensity of the magnetic field are set to 100 kHz and 200 Oe, respectively. Once being irradiated, the temperature of suspension was measured by an alcohol thermometer at 60 s intervals.

### Cell culture and cytotoxicity assay

Human epidermal keratinocyte (HaCaT) cells were purchased from China Center for Type Culture Collection (Wuhan) and maintained in high glucose Dulbecco's Modified Eagle Medium (DMEM) (Gibco, USA) in a humidified 37 °C incubator (Thermos HERAcell150, USA) at 5% CO_2_ supplemented with 10% fetal bovine serum (FBS) (Gibco, USA) and 1% penicillin–streptomycin (Gibco, USA). After sterilized with 75% (v/v) ethanol/deionized water for 24 h and rinsed with phosphate buffer saline (PBS) three times, the MCNTs were suspended in cell culture medium with a concentration of 0 μg ml^−1^, 6.25 μg ml^−1^, 12.5 μg ml^−1^, 25 μg ml^−1^, 50 μg ml^−1^ and 100 μg ml^−1^. HaCaT cells were seeded in 24-well plates and 96-well plates with a density of 5 × 10^4^ cells per ml for evaluation of *in vitro* cytotoxicity of MCNTs.

The quantitative evaluation of *in vitro* cytotoxicity of MCNTs against HaCaT cells were performed by using the cell counting kit-8 (CCK-8) assay (Dojindo Laboratories Kumamoto, Japan). HaCaT cells were seeded in 96-well plates with a density of 5 × 10^4^ cells per ml. After 3 h of culture, adherent cells were exposed to the fresh culture medium containing 0 μg ml^−1^ (control), 6.25 μg ml^−1^, 12.5 μg ml^−1^, 25 μg ml^−1^, 50 μg ml^−1^ and 100 μg ml^−1^ MCNTs. At 24 h, 48 h and 72 h, the cell viability was assessed. The culture medium was briefly refreshed, and 10 μl CCK-8 was added to each well containing 100 μl new medium. After incubated in dark at 37 °C for 2 h, 100 μl of each MCNTs solution was transferred to a new 96-well plate to measure the absorbance of the formazan product at 450 nm by using a microplate reader (SPECTRAFLUOR, TECAN, Sunrise, Austria). The cell viability was calculated through formula (1):

1 Viability (%) = [OD_nanoparticles_ − OD_blank_]/[OD_control_ − OD_blank_] × 100%

where OD_nanoparticles_ is the optical density of the well containing various concentration of nanoparticles, DMEM medium and cells. OD_control_ is the optical density of the well containing DMEM medium and cells. OD_blank_ is the optical density of the well only containing DMEM medium.

In addition, cell viability was observed by staining with Calcein-AM, Hochest 33258, and propidium iodide (PI). After 72 h of culture, cells were rinsed with PBS for three times and stained with PBS containing 2 μmol L^−1^ Calcein-AM, 5 μg ml^−1^ Hochest 33258, and 4 μmol L^−1^ PI (Sigma, Mo, USA). After incubated in dark at 37 °C for 15 min, cells were rinsed with PBS for three times. Then the viable and dead cells were imaged using a fluorescent microscope (OLYMPUS BX71, Japan).

## Results and discussion

**Fig. 2:**
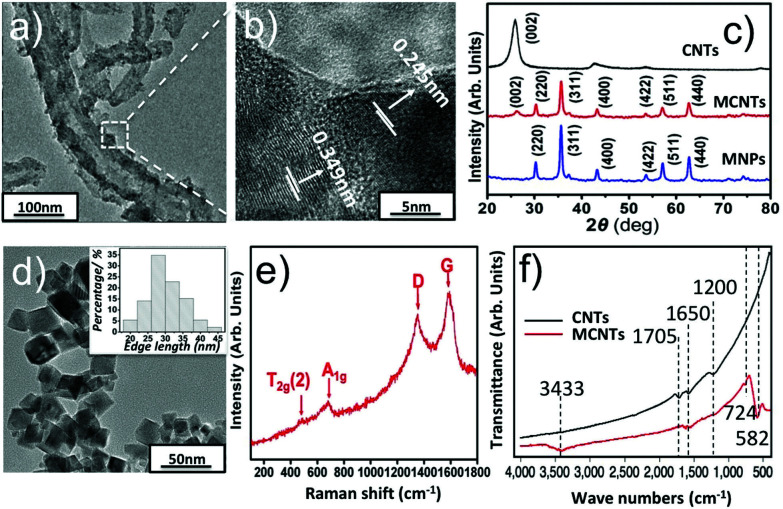
(a) TEM image of MCNTs; (b) HRTEM image of MCNTs; (c) XRD patterns of carboxylic CNTs, MCNTs and MNPs; (d) TEM image of MNPs; (e) Raman spectra of MCNTs; (f) FTIR spectra of MCNTs and carboxylic CNTs.

The size, morphology and crystalline information have been investigated by TEM, XRD, Raman and FTIR analysis, given in Fig. 2. Fig. 2a and d show the representative TEM images of MCNTs and MNPs respectively. Inset of Fig. 2d shows the size distribution of Zn_0.54_Co_0.46_Cr_0.6_Fe_1.4_O_4_ nanoparticles, which is obtained from 100 nanoparticles. The mean edge length of the nanoparticles is found to be 30.3 nm. After adding CNTs, the nanoparticles were successfully decorated on the surface of CNTs. To identify the actual concentration of Zn_0.54_Co_0.46_Cr_0.6_Fe_1.4_O_4_ in MCNTs, we heated MCNTs at 800 °C for 2 h in air to remove CNTs and then the weight percent of Zn_0.54_Co_0.46_Cr_0.6_Fe_1.4_O_4_ in MCNTs is calculated, which is around 73.5%. To obtain the detailed crystalline information of MCNTs, HRTEM and XRD were performed. Fig. 2b shows the interlayer distances of MCNTs are 0.245 nm and 0.349 nm, corresponding to the lattice planes (311) in spinel nanoparticles and (002) in CNTs.

Fig. 2c shows the XRD phase analysis. Patterns of MNPs can be easily indexed to cubic spinel structure of CoFe_2_O_4_–CoCrFe_2_O_4_ (JCPDS-ICDD, 03-0864); and no obvious XRD peaks from impurities could be observed. Compared with MNPs, the patterns of MCNTs have an extra peak (002) arising from CNTs.^28^ The element information was determined by an Electron-Probe Microanalyzer. The results, together with the molar ratio of metal elements initially added, are given in Table 1s.† Clearly, all metal elements in the samples do not deviate significantly from their initial stoichiometry. Based on the Electron-Probe Microanalyzer and XRD experiments, it could be concluded that the all the metal ions successfully enter the lattice. The Raman spectra of MCNTs are given in Fig. 2e. D and G bands at 1346 cm^−1^ and 1575 cm^−1^ correspond to the sp^3^ and sp^2^ hybridized carbons respectively,^29^ arising from CNTs. Peak at 477 cm^−3^ is assigned to T_2g_(2) mode, corresponding to the local symmetry vibrations of metal ions in the octahedral site of spinel nanoparticles.^30^ Peak at 678 cm^−1^ is related to A_1g_ mode arising from the stretching vibrations of Fe^3+^ and O^2−^ in the tetrahedral site in spinel nanoparticles.^31^ These active vibration modes show the spinel structure of nanoparticles attached on CNTs. A combination of TEM, XRD and Raman spectra analyses confirm the crystalline structure of MCNTs.

To have a clear observation of the changes in functional groups on MCNTs, FTIR was performed. As shown in Fig. 2f, compared with the spectra of carboxylic CNTs, the band peaks at 582 cm^−1^, 3433 cm^−1^ appeared and peaks at 1200 cm^−1^, 1705 cm^−1^ vanished on MCNTs. The appearance of peaks at 582 cm^−1^ could be assigned to the stretching vibration of the metal–oxygen bond at tetrahedral site in spinel structure of Zn_0.54_Co_0.46_Cr_0.6_Fe_1.4_O_4_ nanoparticles, formed on the surface of CNTs.^32^ The appearance of a broad peak at 3433 cm^−1^ indicates the existence of hydroxyl group on MCNTs, which may be induced from alkaline solution during the hydrothermal process.^33^ The bands at 1200 cm^−1^, 1705 cm^−1^ and 724 cm^−1^ are all attributed to the carboxylic group on CNTs, corresponding to the bending of C–O bond, stretching and bending vibration of C=O bond respectively. Compared with CNTs, the disappearance of 1705 cm^−1^, 1200 cm^−1^ and the appearance of 724 cm^−1^ on MCNTs shows change of carboxylic group, which mainly results from the formation of either a monodentate complex or a bidentate complex between the carboxyl group and metal atoms on the surface of nanoparticles.^34^ These spectroscopic changes support that the nanoparticles are covalently bound to the carboxylic CNTs.

**Fig. 3:**
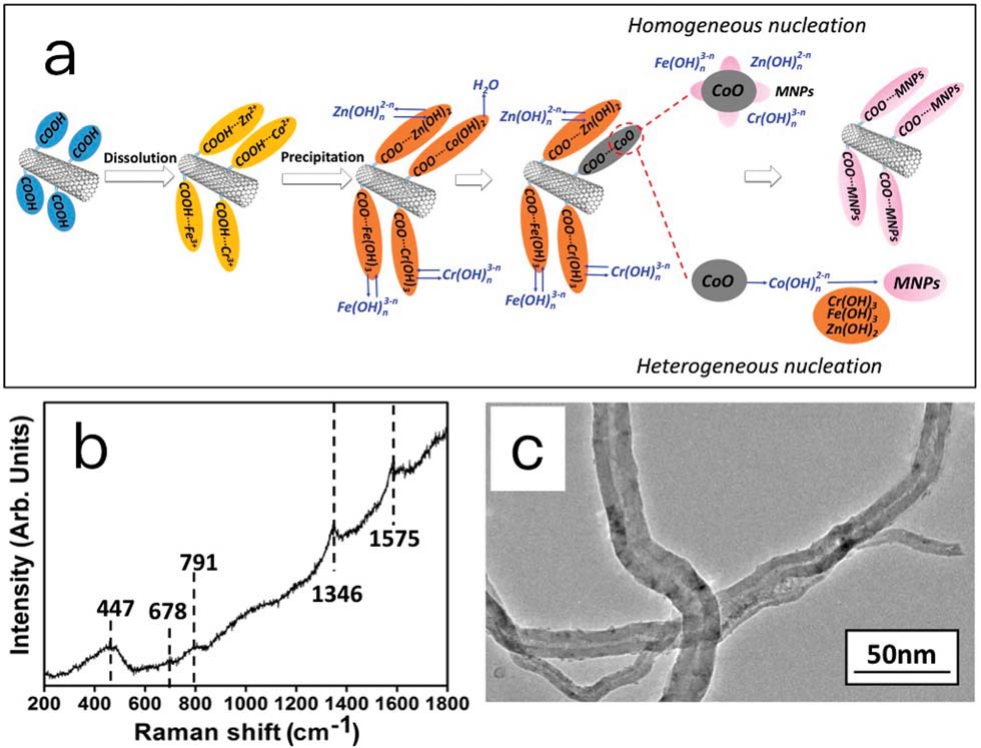
Formation mechanism of MCNTs. (a) Proposed formation mechanism of MCNTs; (b) Raman spectra of precursor MCNTs taken at 100 °C; (c) TEM image of magnetic carbon nanotubes prepared using pristine carbon nanotubes.

The formation mechanism of MCNTs was investigated and proposed, as depicted in Fig. 3a. The carboxyl groups on the surface of CNTs provide sites for the nucleation and growth of nanoparticles. In this experiment, after dissolving the carboxylic CNTs into metal salt solution, the metal ions (Zn^2+^, Co^2+^, Fe^3+^ and Cr^3+^) are preferentially absorbed on these sites due to the electrostatic force. When a NaOH solution is added, the precursors, metal hydroxides attached on the CNTs, form. With increasing temperature, dehydration reactions take place on Co(OH)_2_ and yields crystallized CoO. This CoO serves as the basis for the formation of Zn_0.54_Co_0.46_Cr_0.6_Fe_1.4_O_4_ nanoparticles on CNTs by either heterogeneous nucleation or homogeneous nucleation. In the heterogeneous nucleation pathway, ions or ionomers such as Fe (OH)_*n*_^3−*n*^, Cr(OH)_*n*_^3−*n*^ and Zn(OH)_*n*_^2−*n*^ dissolve from the hydroxide and adsorb onto the surface of crystal CoO. Crystalline spinel Zn_0.54_Co_0.46_Cr_0.6_Fe_1.4_O_4_ forms on the surface of crystal CoO through dehydration and molecular rearrangement. Within the homogeneous nucleation pathway, the Co–O bond may break due to the attack of H_2_O molecules, and form a complex cobalt ion Co(OH)_*n*_^2−*n*^ and dissolve into the solution. The Fe(OH)_3_, Cr(OH)_3_ and Zn(OH)_2_ attached on the CNTs then react with Co(OH)_*n*_^2−*n*^ and forms Zn_0.54_Co_0.46_Cr_0.6_Fe_1.4_O_4_ crystals. In this proposed mechanism, the generation of CoO and the existence of carboxylic groups on CNTs are essential.

To obtain evidence for the proposed mechanisms, two experiments were conducted. Raman spectroscopy is the first one to characterize the structure of precursor taken at 100 °C. The result is given in Fig. 3b. Raman modes at 1346 and 1575 cm^−1^ could be attributed to the D band and G band of CNTs. The other three Raman modes at 791, 678 and 447 cm^−1^ are assigned to CoOOH, Zn_0.54_Co_0.46_Cr_0.6_Fe_1.4_O_4_ and CoO, respectively.^35^ In the Raman spectra of MCNTs (Fig. 2e), however, the peaks of CoO and CoOOH disappeared, and the peaks of Zn_0.54_Co_0.46_Cr_0.6_Fe_1.4_O_4_ are enhanced. This supports the aforementioned mechanism in that the CoO, Zn_0.54_Co_0.46_Cr_0.6_Fe_1.4_O_4_ and Co(OH)_*n*_^2−*n*^ coexist in the reaction system, and finally CoO and Co(OH)_*n*_^2−*n*^ are all transformed into Zn_0.54_Co_0.46_Cr_0.6_Fe_1.4_O_4_.

In the second experiment, pristine multi-walled carbon nanotubes without any functional groups were used to prepared magnetic carbon nanotubes. Fig. 3c shows the TEM image of magnetic carbon nanotubes. No magnetic nanoparticles were found attached on the surface of carbon nanotubes. This supports the proposed mechanism that carboxylic groups provide sites for the nucleation and growth of nanoparticles due to the electrostatic force, and nanoparticles cannot attach on the surface of carbon nanotubes without carboxylic groups.

**Fig. 4:**
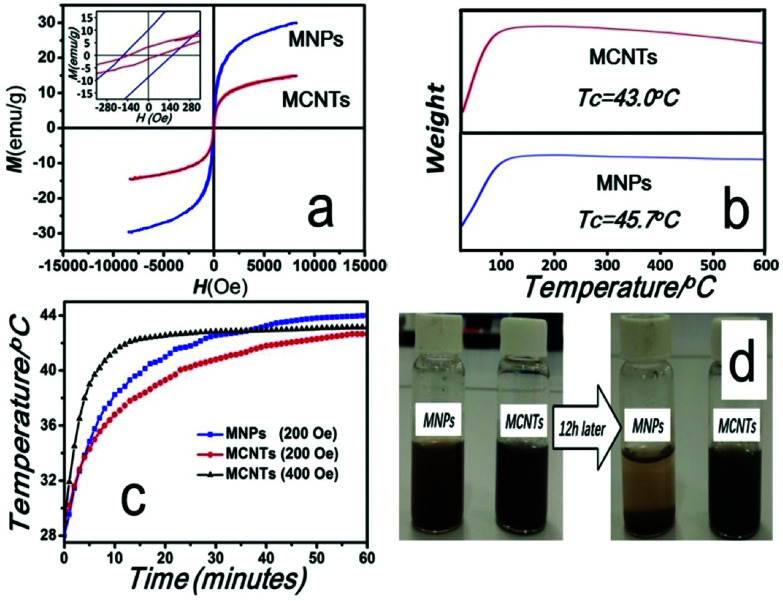
Magnetic properties and dispersibility of MCNTs and MNPs. (a) Magnetization curves of MNPs and MCNTs. The *H*_c_ and *M*_s_ of MCNTs are 129 Oe and 15 emu g^−1^ respectively, which are lower than that of MNPs (172 Oe and 34 emu g^−1^). (b) Thermogravimetric curves of MCNTs and MNPs. The Curie temperature of MNPs and MCNTs are 45.7 °C and 43.0 °C respectively. (c) Time-dependent temperature curves of MNPs and MCNTs in 100 kHz alternating magnetic field (blue line and red line: 200 Oe; Black line: 400 Oe). MCNTs with Curie temperature at 43 °C result in the self-regulating temperature of MCNTs suspension at hyperthermia temperature (around 43 °C). (d) MNPs and MCNTs dispersed in aqueous solution.

The magnetic properties of MNPs and MCNTs were displayed in Fig. 4. Room temperature (300 K) magnetization hysteresis loops were shown in Fig. 4a. The coercivity (*H*_c_) and saturation magnetization (*M*_s_) of MCNTs are 129 Oe and 15 emu g^−1^ respectively, which is lower than that of MNPs (172 Oe and 34 emu g^−1^). The difference of *H*_c_ and *M*_s_ mainly attributes the addition of nonmagnetic CNTs, which decrease the magnetic moment per unit mass.

The thermogravimetric curves are shown in Fig. 4b. As the temperature increases, the nominal weight of MNPs and MCNTs both increase initially, which means that the magnetization decrease with increasing temperature. When the temperature exceeds the Curie temperature, the nominal weight will not change. It is also observed that the temperature range of losing magnetization is broad. This is caused by the individual size differences of nanoparticles. According to Nikolaev's research,^36^ the Curie temperature is directly proportional to the bulk density of exchange bonds. The thickness of surface layer increases with decreasing particle radius. Since the number of exchange bonds at the surface layer is less than that at the particle core, the smaller the nanoparticle size, the lower the Curie temperature. Thus, the temperature range of losing magnetization may be influenced by the size distribution of nanoparticles.

When the temperature reaches the Curie point of a majority of nanoparticles, it experiences the most rapid weight increase in the curves, and the Curie temperature can be determined by taking the maximum value of the first derivative of the thermogravimetric curves. The Curie points of MNPs and MCNTs are decided as 45.7 °C and 43.0 °C respectively. Compared with MNPs, the TGA curves of MCNTs shows a slight thermal degradation when the temperature increases over 200 °C. This weight loss is mainly attributed to the decarboxylation of the carboxylic groups present on the CNTs.^37^

The time-dependent temperature curves of MNPs and MCNTs suspensions were determined by calorimetric measurements. MNPs and MCNTs were dispersed into deionized water with the same concentration (112 mg ml^−1^) before exposure to the external alternating magnetic fields (200 Oe, 100 kHz), which is applicable to clinical therapy.^26,27^ As can be seen in Fig. 4c (blue line and red line), after being irradiated for 60 min, the temperature of suspension could rise to 44.0 °C and 42.7 °C respectively. In order to evaluate heat efficiency quantitatively, specific absorption rate (SAR) under alternating magnetic fields, which describes the energy converted into heat per time and mass, was calculated using the formula (2):^38^

2 SAR = *C*(d*T*/d*t*)(*m*_s_/*m*_m_)

where *C* is the specific heat capacity of suspension (4.18 J gK^−1^); d*T*/d*t* is the slope of the time-dependent temperature curve; *m*_s_ is the mass of suspension and *m*_m_ is the mass of magnetic media in the suspension. As the temperature higher than room temperature will cause heat loss to surroundings which may significantly influence value of SAR,^39,40^ here we calculated SAR just at room temperature and select d*T*/d*t* at the initial several seconds of the experiment, when heat transfer to surroundings can be ignored. The SAR of MNPs and MCNTs are 774 W kg^−1^ and 695 W kg^−1^ respectively. As the *H*_c_ and *M*_s_ of MCNTs are lower than that of MNPs, the area of hysteresis loop of MCNTs is smaller than that of MNPs, which result in lower heat efficiency of MCNTs.

After a rapid increase of temperature, the MNPs and MCNTs suspensions reach a stable temperature of 44.0 °C and 42.7 °C, which is close to their Curie temperatures (45.7 °C and 43.0 °C). To investigate the ability of self-regulating temperature of MCNTs, additional magnetic heating experiment with higher SAR has been conducted. Here, time-dependent temperature curve under the magnetic field of 400 Oe is recorded and the result is given in Fig. 4c (black line). Although SAR is much higher (1372 W kg^−1^), MCNTs suspensions reach the stable temperature of 43.1 °C, still fairly close to the stable temperature under 200 Oe and Curie temperature. Only when the hyperthermia temperature is maintained below the Curie point, MCNTs keeps their magnetism and generate sufficient heat under an alternating magnetic field in this self-regulating system. Otherwise, the magnetic media will lose their magnetism and not generate heat, which makes MCNTs suspension remain constant around its Curie temperature. These experiments demonstrate the self-regulating nature of MCNTs and self-regulating temperature around 43 °C, meet the requirement for hyperthermia.

Fig. 4d shows the dispersed states of MNPs and MCNTs in aqueous media at the moment of dispersion and after ∼12 h. Most MNPs precipitated after 12 h while MCNTs are still finely dispersed. The UV-vis spectra were performed to monitor dispersion of MNPs and MCNTs in aqueous media and the results are given in Fig. 1s.† The absorbance peak of MNPs decreased 65.2% after sedimentation for 12 h, which is almost 5 times higher than MCNTs. Compared with MNPs, MCNTs have a better dispersibility. MNPs with great surface energy and the magnetic force have strong tendency to aggregate together. This is because the gravity of aggregate is stronger than the dispersive effect caused by Brownian motion. For MCNTs, the carboxylic groups on nanotube surface are negatively charged, which enable the MCNTs to repel from each other and keep the solution dispersed. MCNTs with a better dispersibility may avoid the inhomogeneous temperature distribution caused by the aggregation of MNPs in magnetic hyperthermia.

Another concern about the medical application is the biocompatibility of MCNTs. In clinical trials, nanoparticles are injected into tumor regions and most of them are allocated in tumor tissue, which resulted in the several times higher uptake of nanoparticles in tumor than that in normal tissue. Only 2.7–10% nanoparticles could be detected in other normal tissue even after 10 days' treatment.^41,42^ After blood circulation and metabolism, the concentration of nanoparticles stay in normal tissue is low. For many cytotoxicity studies of hyperthermia, 100 μg ml^−1^ is considered as a very high concentration of nanoparticles that can extravasate into normal tissue.^43,44^ Therefore, here we also select 100 μg ml^−1^ for cytotoxicity studies.

**Fig. 5:**
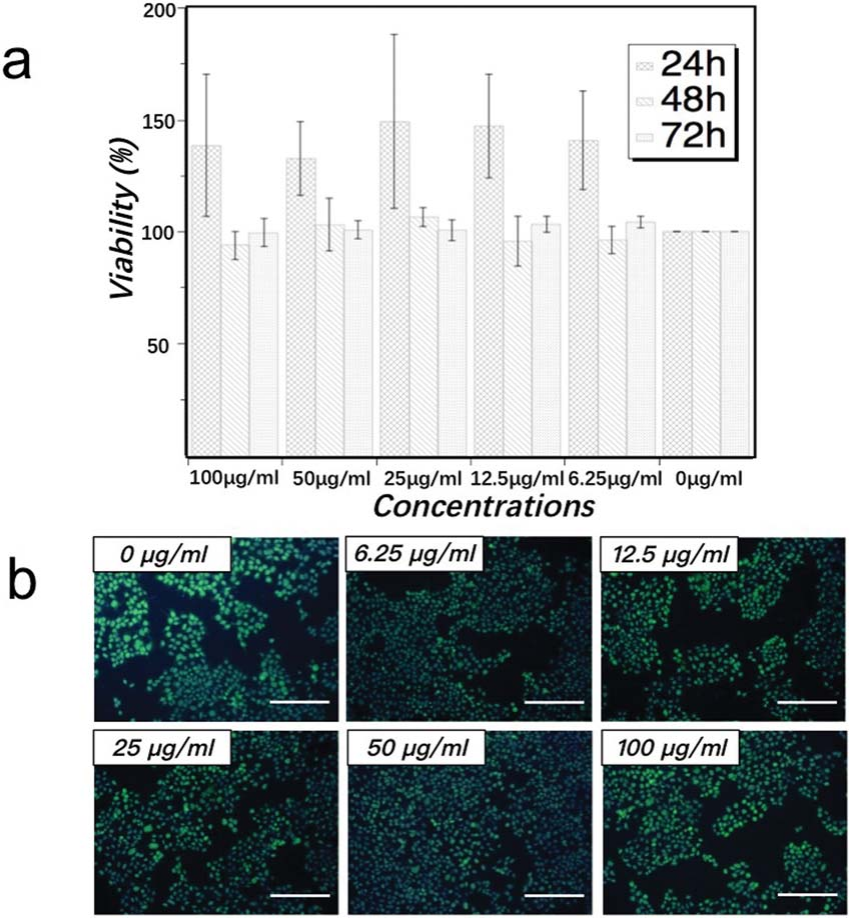
Effects of MCNTs on the viability of HaCaT cells: (a) cell viability was assessed by CCK-8 assay at 24 h, 48 h and 72 h after co-culture with various concentrations of MCNTs. Values were represented as the percentages of cell viability compared with that of the control group and expressed as means ± standard deviation of triplicate determinations. (b) Representative fluorescent images of detached HaCaT cells which are triple stained with Calcein-AM (green), PI (red) and Hochest (blue) after co-culture with various concentrations of MCNTs for 72 h. Scale bar: 50 μm.

The cytotoxicity effect of MCNTs with the concentrations of 0 μg ml^−1^, 6.25 μg ml^−1^, 12.5 μg ml^−1^, 25 μg ml^−1^, 50 μg ml^−1^ and 100 μg ml^−1^ on HaCaT cells was examined after co-culture of 24 h, 48 h and 72 h. As shown in Fig. 5a, the viability of HaCaT cells in all MCNTs concentrations are above 85%, and there is no significant difference in cell viability between all MCNTs groups and control group (*p*-value > 0.05) after co-culture of 48 h and 72 h. After co-culture of 24 h, all MCNTs groups show some enhancement of cell viability. The similar phenomenon has also been observed in the cytotoxicity assay of gold nanoparticles and the enhanced growth of cell may be related to the receptor-mediated control of cell membranes.^45,46^ The more detailed mechanism is still under investigation.

In order to have more visualized observation of cell state, live-dead fluorescent microscopic analysis was performed. After co-culture with MCNTs for 72 h, cells were stained with Calcein-AM, PI and Hochest 33258. Dead cells can be identified by a red fluorescence generated by PI after intercalating into DNA, which only occurs after the entrance of the dead cell-permeable dye to the cell membrane. Cell nucleus can be identified by a blue fluorescence generated by Hochest 33258 after the binding of this living cell-permeable dye to DNA. Living cells can be identified by a green fluorescence generated by the enzymatic hydrolysis of Calcein-AM, which only takes place in living cells as a result of esterase activity. As can be seen in Fig. 5b, cells co-cultured with all concentrations of MCNTs for 72 h show calcein-positive, Hochest 33258-positive and PI-negative state. This live-dead result is consistent with the quantitative CCK-8 assay in Fig. 5a. These results show that the MCNTs have low cytotoxicity for HaCaT *in vitro* under the concentrations of 6.25 μg ml^−1^ to 100 μg ml^−1^. As cytotoxicity test is a quite complex systematic work, we will continue in-depth studies for other cell lines.

## Conclusions

Magnetic carbon nanotubes with a Curie temperature of 45.7 °C for self-regulating temperature hyperthermia were prepared through combining carboxyl carbon nanotubes and nanoparticles using hydrothermal method. Nanoparticles were successfully decorated on the surface of CNTs. The carboxylic groups on CNTs and the formation and hydration of CoO played important roles in the mechanisms of MCNTs formation, heterogeneous or homogeneous nucleation processes. MCNTs also show a better dispersibility than MNPs, which may avoid the inhomogeneous temperature distribution caused by the aggregation of nanoparticles in magnetic hyperthermia. The Curie temperature of MCNTs is 43 °C while the temperature of MCNTs suspension can be self-regulated at 42.7 °C under the clinically used magnetic fields, which is suitable for self-regulating temperature hyperthermia. The evaluation of *in vitro* cytotoxicity of MCNTs also suggests low toxicity under the concentrations of 6.25 μg ml^−1^ to 100 μg ml^−1^. This study proposed a methodology on the bespoke synthesis of magnetic carbon nanotube for self-regulating hyperthermia, which may lead to further research on loading drugs for multi-modal cancer therapy.

## Conflicts of interest

There are no conflicts to declare.

## Supplementary Material

RA-008-C7RA13256E-s001
