# Supplementary material for: Magnetic carbon nanotubes for self-regulating temperature hyperthermia
Source: RSC Adv. 2018 Mar 27;8(22):11997–2003. doi: 10.1039/c7ra13256e (PMC9079272; doi:10.1039/c7ra13256e)
Supplement: RA-008-C7RA13256E-s001 [file RA-008-C7RA13256E-s001.pdf]

Electronic Supplementary Material (ESI) for RSC Advances.
This journal is © The Royal Society of Chemistry 2018

Supporting Information

Magnetic carbon nanotubes for self-regulating temperature hyperthermia †

Xudong Zuo,[a] Chengwei Wu,[a] Wei Zhang *[a] and Wei Gao[b]

Table 1s The theoretical and measured element ratios of MNPs and MCNTs

Sample name	Theoretical molar ratio (Zn:Co:Cr:Fe)	Measured molar ratio (Zn:Co:Cr:Fe)
MNPs	0.54:0.46:0.6:1.4	0.539:0.461:0.54:1.46
MCNTs	0.54:0.46:0.6:1.4	0.545:0.455:0.56:1.44

[a]State Key Laboratory of Structural Analysis for Industrial Equipment, Department of Engineering Mechanics, Dalian University of Technology, Dalian 116024, P. R. China. Email: wei.zhang@dlut.edu.cn

[b]Department of Chemical and Materials Engineering, the University of Auckland, Auckland 1142, New Zealand.

†Electronic supplementary information available: Table S1-The theoretical and measured element ratios of MNPs and MCNTs, Figure S1- UV-vis spectra of MNPs and MCNTs aqueous solution at the moment of dispersion and after 12 h.

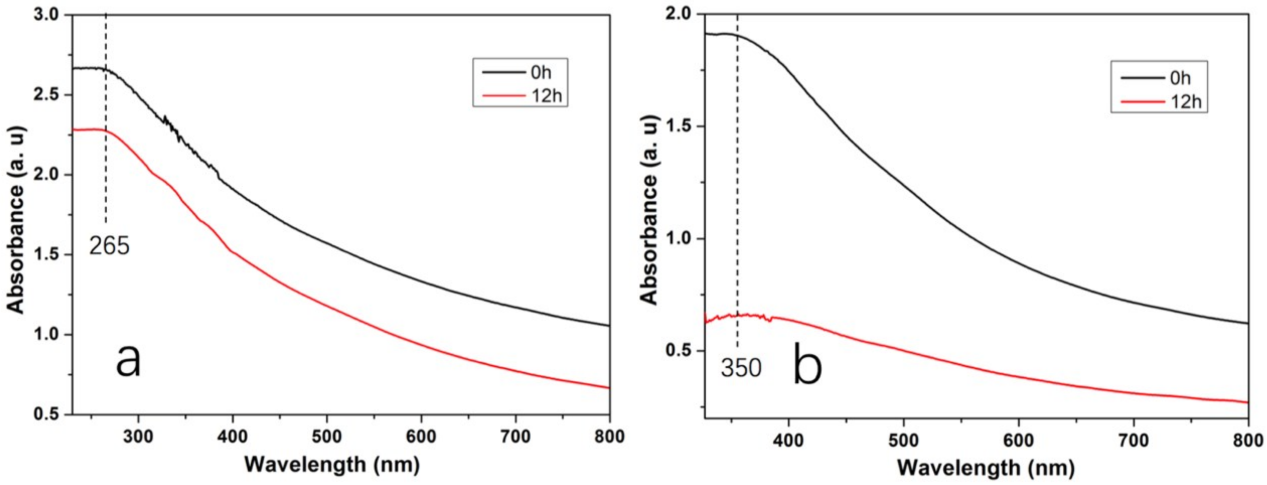

Figure 1s (a) UV-vis spectra of MCNTs aqueous solution at the moment of dispersion and after 12 h; (b) UV-vis spectra of MNPs aqueous solution at the moment of dispersion and after 12 h.
